# Recovery of Protein from Industrial Hemp Waste (*Cannabis sativa*, L.) Using High-Pressure Processing and Ultrasound Technologies

**DOI:** 10.3390/foods12152883

**Published:** 2023-07-29

**Authors:** Eduarda M. Cabral, Xianglu Zhu, Marco Garcia-Vaquero, Sara Pérez-Vila, Jiafei Tang, Laura G. Gómez-Mascaraque, Mahesha M. Poojary, James Curtin, Brijesh K. Tiwari

**Affiliations:** 1 Department of Food Quality and Sensory Science, Teagasc Ashtown Food Research Centre, Dublin 15, Ireland; 2 Department of Food Chemistry and Technology, Teagasc Ashtown Food Research Centre, Dublin 15, Ireland; 3 School of Biosystems and Food Engineering, University College Dublin, Dublin 4, Ireland; 4 School of Agriculture and Food Science, University College Dublin, Dublin 4, Ireland; 5 Department of Food Chemistry and Technology, Teagasc Moorepark Food Research Centre, co. Cork, P61 C996, Ireland; 6 School of Food and Nutritional Sciences, University College Cork, Cork, T12 K8AF, Ireland; 7 Department of Food Science, Faculty of Science, University of Copenhagen, 1958 Frederiksberg C, Denmark; 8 School of Food Science & Environmental Health, College of Sciences & Health, Technological University Dublin, Park House Grangegorman, 191 North Circular Road, Dublin 7, Ireland

**Keywords:** circular bioeconomy, green process, hempseed cake, high-pressure processing, ultrasound-assisted extraction, hemp protein isolate, hemp protein recovery

## Abstract

Hemp seeds are currently used mainly for oil extraction, generating waste that could be potentially exploited further as a source of proteins and other bioactives. This study aims to valorise hemp waste (*Cannabis sativa*, L.) from previous oil extraction as a source of protein by analysing the effect of high-pressure processing (HPP) pre-treatments (0–600 MPa; 4–8 min) combined with conventional or ultrasound-assisted extraction (UAE) methods on protein recovery/purity, amino acid composition, and protein structure. Overall, maximum protein recovery (≈62%) was achieved with HPP (200 MPa, 8 min) with UAE. The highest protein purity (≈76%) was achieved with HPP (200 MPa, 4 min) with UAE. Overall, UAE improved the extraction of all amino acids compared to conventional extraction independently of HPP pre-treatments. Arg/Lys ratios of the protein isolates ranged between 3.78 and 5.34, higher than other vegetable protein sources. SDS-PAGE did not show visible differences amongst the protein isolates. These results seem to indicate the advantages of the use of UAE for protein recovery in the food industry and the need for further studies to optimise HPP/UAE for an accurate estimation of processing costs and their effects on the composition and structure of proteins to contribute further to the circular economy.

## 1. Introduction

It is highly beneficial, both economically and from a social and environmental standpoint, to invest in initiatives aimed at reducing food waste and discovering innovations that maximize the value of discarded food. The current economic model based on the take–make–dispose criterion is becoming environmentally unsustainable. This exploitation model of resources has negative effects on global ecosystems as it can lead to over-exploitation of natural resources and environmental degradation; the agri-food sector is widely affected by these issues [1]. Therefore, it is relevant to have in place efficient resources and/or waste management programmes aiming to further utilise resources following the concept of “waste = food” or circular exploitation models that contribute to a circular economy. In fact, the use of waste for the recovery of “food value” is considered one of the pillars of food waste and food waste management within the circular economy in the food sector [2]. One particular strategy gaining significant traction in the food production and manufacturing industries is the use of by-products and food waste as biomass for the recovery of high-value ingredients, on the basis of their favourable nutritional composition and biological properties, that could be re-introduced in the food chain via the formulation of novel foods, contributing to increasing in this way the circularity of the industrial processes and use of resources of the traditional agri-food production systems, as well as adding further economic and societal value. One sustainable solution for the reduction and utilisation of food waste is the extraction of its valuable compounds, such as protein and other compounds, from under-utilised resources [2].

Within this scenario, seeds from hemp (*Cannabis sativa*, L.) are commonly used for oil extraction generating defatted hempseed waste, a clear example of a valuable and underutilized industrial waste material that can be an excellent source of proteins, amino acids, and other valuable bioactive compounds with promising health applications [3,4,5]. In fact, hemp cake has been described as a good source of histidine and albumin proteins [3] with promising potential for the production of protein isolates (>90% protein content) and concentrates (30–80% protein). The extraction, fractionation, and recovery of bioactive compounds can be affected by several factors, such as the type of raw materials, solvents, and the technological processing of biomass for the extraction of the compounds [6]. These processing techniques are often classified as conventional methods, which are normally inefficient in terms of treatment times and using large amounts of solvents, and as innovative technologies, such as high-pressure processing (HPP) and ultrasound-assisted extraction (UAE), with promising results to develop efficient processes aiming to achieve greener technological processing solutions for multiple applications, contributing further to the circular economy by means of decreased resource utilisation [6,7]. Thereby, HPP has been recently explored for its ability to increase extraction of bioactives by various phenomena (i.e., changes in reaction dynamics, cell structures, etc.) useful when extracting protein ingredients from multiple sources [8]. UAE is based on the application of mechanical waves with a frequency higher than that of the audible range for humans (>20 kHz) that generate compressions and rarefaction responsible for inducing significant physical and chemical effects in food processing during extraction processes [7]. Overall, the advantages of these innovative techniques include shorter processing times, better extraction yields, lower negative impact on the activity and structure of the bioactive compounds, and lower energy consumption compared with conventional methods [6,7]. Moreover, recent trends in food technology include the sequential and/or simultaneous application of several extraction forces by means of innovative technologies as a way to improve the efficiency of the processes of extraction of high-value compounds even further [9,10]. To the best of our knowledge, the vast majority of the scientific literature focuses on the use of multiple innovative technologies to improve the extraction of oil, cannabinoids, and other lipophilic compounds from intact hemp seeds [11,12,13,14] and their biological properties, including antimicrobial [15], anti-obesity [16] and antioxidant [13] amongst others. However, limited information is currently available on the valorisation strategies of hemp waste from oil extraction by recovering proteins and other bioactives [17,18,19], and to our knowledge, there are no reports exploring the sequential application of HPP and UAE innovative technologies and their effect on the final protein isolates. Information about the applicability of HPP and UAE as green technologies when processing hempseed protein and other high-value products from hempseed is limited and can provide useful insights for the future use of these processes at the industrial level when recovering high-value products with nutritional values of great interest for the food and nutraceutical industries.

This study aims to establish the potential of an industrial waste product, defatted hempseed cake (*Cannabis sativa*, L.), as a source of protein ingredients. The effects of HPP pre-treatment conditions, pressure (200, 400, and 600 MPa), and time (4 and 8 min), followed by either conventional or UAE extraction methods on the protein recovery and protein purity of protein isolates generated by isoelectric precipitation were also explored. The influence of different technological processing conditions on the amino acid composition, including essential, conditionally essential, and non-essential amino acids, as well as on the protein structure of the protein isolates, was also determined, aiming to establish a future model for the exploitation of this biomass waste by applying innovative and green processing technologies to contribute to the circular economy.

## 2. Materials and Methods

### 2.1. Raw Material and Chemicals

A local Irish farmer (Co. Kildare, Ireland) provided defatted hempseed cake following a cold oil extraction process. The hempseed cake pellets were ground (Lloytron E5012WI, Kitchen Perfected Blender, Leigh, UK), vacuum-packed, and preserved under refrigeration conditions (4 °C) until the application of further technological processing or chemical analyses.

All the chemicals used in this study were of analytical grade. The reagents used during the technological processing of the biomass include sodium hydroxide (NaOH) purchased from Sigma-Aldrich (St. Louis, MO, USA), hydrochloric acid (HCl, 37 % *w*/*v*) purchased from Honeywell-Fluka (Austria), and ultrapure water (Milli-Q System, Millipore, Burlington, MA, USA). For amino acid analysis, an amino acid calibration standard mix was purchased from Sigma Aldrich (Copenhagen, Denmark), and HPLC grade acetonitrile and methanol were obtained from VWR International (Søborg, Denmark). Other materials and reagents for SDS-PAGE analyses include NuPAGE^®^ LDS sample buffer, NuPAGE^®^ sample reducing agent, NuPAGE^®^ 4–12% Bis-Tris Gel 1.0 mm, NuPAGE^®^ antioxidant (Invitrogen, Life Technologies Corp., Waltham, CA, USA), PageRuler pre-stained protein ladder (Thermo Scientific, Vilnius, Lithuania), and Ready BlueTM (Sigma-Aldrich, Darmstadt, Germany).

### 2.2. Technological Processing of Defatted Hempseed Cake

**Figure 1:**
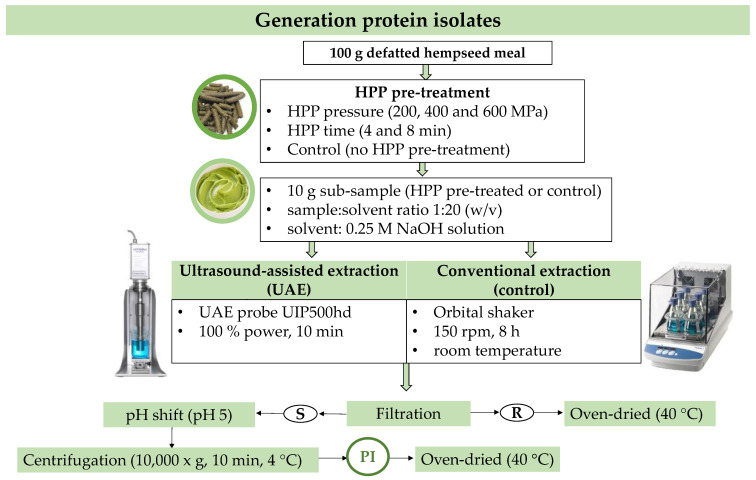
Workflow diagram for the generation of protein isolates from defatted hempseed meal. Abbreviations in the figure are as follows: HPP (high-pressure processing), S (supernatant), R (residue), and PI (protein isolate).

Defatted and ground hempseed samples were processed further to recover protein. The influence of technological pre-treatments using different high-pressure processing (HPP) conditions (200–600 MPa of pressure and 4–8 min of time) or control (no HPP) followed by the application of further extraction technologies (ultrasound-assisted extraction (UAE) or conventional methods) for the generation of protein isolates using pH shift methods is described in detail in Figure 1.

#### 2.2.1. HPP Pre-Treatments

Samples undergoing HPP pre-treatments were prepared as follows. Hempseed cake (100 g) was soaked overnight in Milli-Q water (100 mL), vacuum-packed, and stored at 4 °C until receiving an HPP pre-treatment using a Hiperbaric 420 system (Burgos, Spain) at HPP Tolling (St. Margaret’s, Co., Dublin, Ireland). The HPP pre-treatments were performed at variable pressure conditions (200, 400, and 600 MPa) for either 4 or 8 min. Control experiments were also performed with samples of pre-packed biomass not receiving HPP pre-treatments.

#### 2.2.2. Extraction Technologies

Sub-samples from those receiving HPP pre-treatments or control conditions generated in 2.2.1 were further extracted following either ultrasound-assisted extraction (UAE) or a conventional extraction method. Sub-samples of pre-treated biomass (10 g) were mixed in 0.25 M NaOH solutions at a ratio of biomass:solvent of 1:20 (*w*/*v*). These sub-samples were extracted by either (1) UAE, using an ultrasonic probe UIP500hd (Hielscher Ultrasonics, Teltow, Germany) at 100% power for 10 min, or (2) conventional extraction, using the orbital shaker–incubator Max Q™6000 (Thermo Fisher Scientific, Waltham, MA, USA) at 150 rpm, for 8 h at room temperature. All the samples following UAE and conventional extraction experiments were filtered for the recovery of the supernatants that were kept refrigerated (4 °C) for the generation of protein isolates. The pellets were also collected, oven-dried (40 °C), vacuum-packed, and stored at 4 °C for further analysis. 

#### 2.2.3. Generation of Hemp Protein Isolates

Protein isolates were generated by the precipitation of proteins from all the supernatants after the extraction process following a pH shift method as described by C.-H. Tang et al. [20]. Briefly, the pH of the supernatants was adjusted to 5 by adding 0.3 N HCl, and the protein isolates were collected by centrifugation (10,000× *g*, 10 min), oven-dried, and stored at 4 °C for further chemical analyses.

### 2.3. Chemical Analyses

All chemical analyses were performed in duplicate.

#### 2.3.1. Proximate Composition Analysis

The proximate composition (moisture, ash, protein, fibre, and fat) of the defatted hempseed cake was determined before the application of any pre-treatment or extraction procedure following official methods of analysis (AOAC). The moisture content of the samples was determined using an infrared moisture analyser (MA37, Sartorius Lab. Instruments, GmbH & Co., Göttingen, Germany). Ash contents were determined in a muffle furnace (550 °C, 6 h) following the AOAC.942.05 method [21]. Fat contents were analysed using the NMR fat analyser (Oracle, CEM Corporation, Charlotte, NC, USA) following the AOAC 2008.06 method [22]. Crude fibre contents were determined following the AOAC method 962.09 [21]. Protein contents of defatted hempseed meal, residues from the extraction, and protein isolates were determined using a nitrogen analyser FP628 (LECO Corp., St. Joseph, MI, USA) using a nitrogen-to-protein conversion factor of 6.25 [23]. The protein recovery (%) and purity were calculated based on the following equations:

$$Protein\;recovery\;\left(\%\right)=\frac{Protein\;content\;of\;extract}{Protein\;content\;of\;biomass}\times 100$$

$$Protein\;purity\;\left(\%\right)=\frac{Protein\;content\;of\;extract}{Weight\;of\;extract}\times 100$$

#### 2.3.2. Total Amino Acid Analysis

A total of 10 mg of hemp protein isolate (HPI) was hydrolysed for 24 h at 110 °C under a nitrogen atmosphere using 3 mL of 4 M methanesulfonic acid containing 0.2% (*w*/*v*) tryptamine. The hydrolysates were neutralised with 4 M NaOH, mixed with an equal volume of internal standard (50 μM 6-aminocaproic acid solution), and filtered using 0.22 μm regenerated cellulose syringe membrane filters. The total amino acid content of the HPI was determined using UHPLC-FLD equipment (Thermo Ultimate 3000 RS, Thermo Scientific, Waltham, MA, USA) with an Agilent AdvanceBio AAA column (100 mm × 3.0 mm × 2.7 μm particle size, Agilent Technologies, USA) at the conditions previously described in Hildebrand et al. [24]. Briefly, 10 mM Na_2_HPO_4_ in 10 mM Na_2_B_4_O_7_ decahydrate at pH 8.2 (mobile phase A) and a mixture of acetonitrile:methanol:water 45:45:10, v:v:v (mobile phase B) were used at a flow rate of 0.62 mL/min following the gradient: 2% B (0–0.35 min), 57% B (0.35–13.4 min), 100% B (13.4–13.5 and 13.5–15.7 min), and 2% B (15.7–15.8 and 15.8–18.0 min). Fluorescence detection with an excitation wavelength of 340 nm and an emission wavelength of 450 nm was used to quantify the amino acids using an internal standard calibration method with authentic amino acid calibration standards. The concentration of amino acids in the samples is expressed as mg of each individual amino acid per g of protein isolate.

Total essential amino acid (EAA) values were calculated by adding the concentrations of His, Ile, Leu, Lys, Met, Phe, Thr, Trp, and Val; conditionally essential amino acid (CEAA) concentrations correspond to Arg, Gly, Pro, and Tyr; non-essential amino acids (NEAA) values were calculated by adding the concentrations of Ala, Asp, Glu, and Ser in the samples; and total amino acid (TAA) values were calculated by adding EAA, CEAA, and NEAA. The ratio EAA/TAA (%) refers to the proportion of essential amino acids to total amino acids.

#### 2.3.3. Sodium Dodecyl Sulphate-Polyacrylamide Gel Electrophoresis (SDS-PAGE)

SDS-PAGE was performed to determine the molecular weight (MW) distribution of the proteins of the HPI. HPI samples were dissolved in water up to a final protein concentration of 3 µg/mL and mixed with 2.5 µL NuPAGE^®^ LDS sample buffer and 1 µL NuPAGE^®^ sample reducing agent (Invitrogen, Life Technologies Corp., Waltham, CA, USA) in order to reduce the disulphide bonds and achieve the separation of protein subunits [25]. Samples were loaded into 12-well precast gels NuPAGE^®^ 4–12% Bis-Tris Gel 1.0 mm (Invitrogen, Life Technologies Corp., Waltham, CA, USA). SDS-PAGE was performed in an XCell SureLock Electrophoresis Cell (Novex, Life Technologies, CA, USA) according to the manufacturer’s instructions using MES SDS running buffer (Novex, Life Technologies, Waltham, CA, USA) and NuPAGE^®^ antioxidant (Invitrogen, Life Technologies Corp., Waltham, CA, USA) to maintain proteins in their reduced state. PageRuler pre-stained protein ladder (Thermo Scientific, Vilnius, Lithuania) with MW ranging from 10–180 kDa were used as reference samples in the first and last lanes of each SDS-PAGE. The electrophoresis was performed at 4 °C, 30 V (20 min), and 100 V (120 min). All the gels were stained with Ready BlueTM (Sigma-Aldrich, Darmstadt, Germany) to allow the visualization of the protein bands.

### 2.4. Statistical Analyses 

The technological processing of the samples was performed in duplicate as well as all the chemical determinations (n = 4). All the data were analysed using SPSS version 27.0. Multivariate general linear models were used to determine the influence of HPP pressure, HPP time, and extraction conditions (conventional extraction and UAE) on protein recovery, protein purity, and amino acid composition of the protein isolates. The differences between groups were further analysed by either Student’s *t*-test or Games–Howell post hoc tests for comparisons between 2 or more than 2 groups, respectively. In all cases, the criterion for statistical significance is *p* < 0.05. The variance in the data set was analysed by principal component analysis (PCA) using direct Varimax rotation with Kaiser normalisation to calculate the expected weight for each component with eigenvalues higher than 1. PCA scatter plots were generated by SPSS version 27.0, while all the other graphics were produced in Microsoft Excel.

## 3. Results and Discussion 

### 3.1. Proximate Composition of Defatted Hempseed Cake

The proximate composition of the original biomass used for further technological processing in this study can be considered as high in fibre and protein and low in lipids, as seen in the proximate results in Table 1. The high levels of protein in the defatted hempseed meal used in this study could indicate the potential to explore this material for the generation of protein-rich ingredients (concentrates or isolates). Shen et al. [26] reported similar protein levels of approximately 33% from non-dehulled hempseed meal. Previous reports on the composition of hempseeds also described this biomass as containing variable levels of oil (25–30% on a dry weight (DW) basis), protein (20–30% DW), and fibre (30–40% DW) depending on the genotype of the hemp and other growing factors affecting the development of the plants [23]. Overall, these results indicate that the defatted hempseed cake of this study can be further processed as a source of protein, as it contains protein levels similar to or even higher than other protein-rich products described in the literature, including quinoa (13%), linseeds (20.9%), and buckwheat seeds (27.8%) [27].

### 3.2. Influence of HPP and UAE on Protein Extraction from Defatted Hempseed Cake

**Figure 2:**
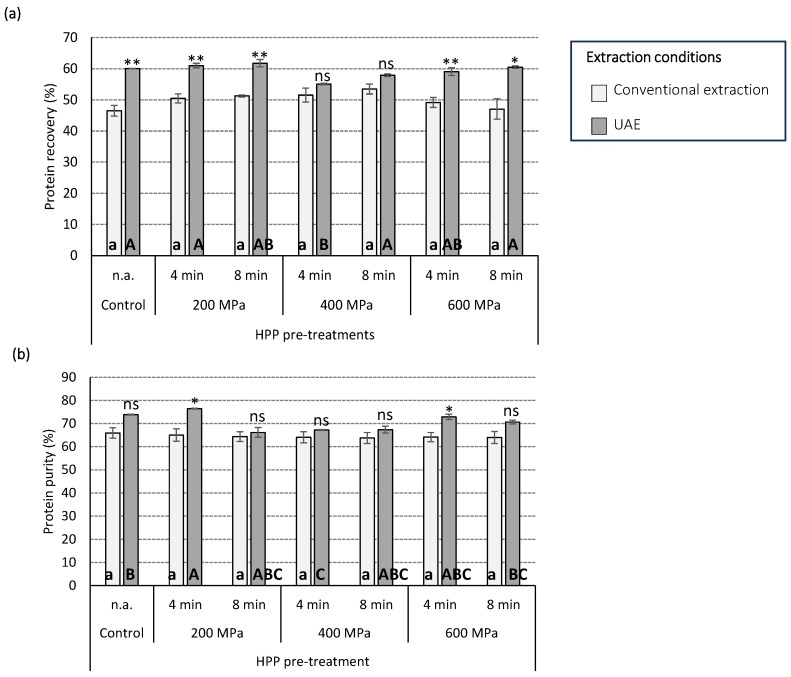
Influence of high-pressure processing (HPP) pre-treatments at variable HPP pressure (200, 400, and 600 MPa), HPP time (4 and 8 min), and extraction conditions (UAE and conventional extraction method) on the (**a**) protein recovery (%) and (**b**) protein purity (%) from defatted hempseed meal. Different lower-case and upper-case letters indicate statistical differences (*p* < 0.05) amongst all the samples extracted following conventional extraction and UAE conditions, respectively. Differences between conventional and UAE methods within each specific HPP-pre-treatment are indicated as * (*p* < 0.05), ** (*p* < 0.01), and ns (not significant).

The influence of the technological processing, including HPP pre-treatment (pressure and time) and UAE or conventional extraction, on the protein recovery and protein purity from defatted hempseed cake is presented in Figure 2a,b. Overall, UAE had a significant influence (*p* < 0.001) on all the parameters analysed, and the interaction HPP pressure*UAE was also significant (*p* < 0.01) in the case of protein recovery. The remaining parameters and interactions between them were not significant for either protein recovery or protein purity.

Overall, the application of any HPP pre-treatment followed by UAE was more efficient when recovering protein from defatted hempseed meal compared to the application of conventional extraction procedures, except in the case of HPP pre-treatments at 400 MPa, which recovered similar levels of protein independently of the extraction method used (Figure 2a). The maximum levels of protein recovery (approximately 62%) were achieved with HPP pre-treatment (200 MPa, 8 min) combined with UAE, while the lowest protein recovery (46%) was achieved by not using HPP pre-treatments followed by conventional extraction methods. Although there is a lack of studies using HPP to recover protein from hemp, these results are in agreement with previous data reporting the effect of HPP as pre-treatment on protein extraction from macroalgae [28]. Suwal et al. [28] explored the recovery of protein from several macroalgal species using HPP followed by enzymatic treatments with polysaccharidases. The authors reported that the application of HPP (400 MPa, 20 min) alone had no significant effect on the recovery of proteins from *Palmaria palmata* and *Solieria chordalis*; the effect of HPP was mainly attributed to the interaction with the enzymatic extraction [28]. The different behaviour of HPP pre-treatment followed by UAE when applied at 400 MPa in this study indicates the need to explore and optimise further the HPP parameters pressure and time, alone or in combination with other extraction technologies, to elucidate the effects of these processing technologies on the biomass. Variable results on protein recovery when applying HPP and with the comparison between the efficiency of HPP and UAE have been described in the literature when exploring several vegetable biomasses. Tang et al. [29] reported that the application of HPP (0–800 MPa, 5 min) alone or as a pre-treatment for enzymatic extraction did not result in a significant improvement in protein recovery from defatted rice bran. However, the authors mentioned that HPP combined with amylase and protease enzymes could have potential and needed further exploration when developing efficient methods to recover protein from defatted rice bran [29]. Preece et al. [30] used high-pressure homogenization (100 MPa) and recovered 82% protein from alkaline soy slurry, while maximum recoveries of 70% were also achieved using an ultrasonic probe at laboratory scale (20 kHz, 65 W, 15 min). Dong et al. [31] reported that pre-treatment with high pressure significantly improved the protein recovery yields from defatted peanut flour, with high protein recovery of approximately 31 and 40% achieved when using 40 MPa and 80 MPa, respectively, as compared to control samples using 0.1 MPa (16.84%). These variable results on protein extraction and recovery yields when using high-pressure treatments could be explained by the variable effects of the processing parameters pressure and time when modifying the surface area of the multiple vegetable biomasses explored, as well as when modifying the microstructure of the different proteins produced by the different biomasses that can influence their extraction [31].

The influence of the technological processing on the protein purity of protein isolates achieved from defatted hempseed cake in this study is presented in Figure 2b. Similar to the results of protein recovery, the main effects of technological processing on protein purity can be attributed to the application of UAE. Overall, the highest purity of protein (76%) was achieved with HPP pre-treatment (200 MPa, 4 min) combined with UAE, while the lowest protein purities (64–66%) were achieved by using any pre-treatment combined with conventional extraction technologies. UAE has been widely reported as a technological treatment to improve the extraction of protein and other phytochemicals from vegetable biomass [7,24,29]. Limited information is available in the scientific literature reporting protein purity. Yang et al. [32] explored the effects of UAE and α-amylase on the protein recovery and purity from rice dreg powder. The authors found the highest levels of protein recovery, approximately 90%, when using ultrasonic frequencies of 20 and 35 kHz combined with the enzymatic treatment. Moreover, extracts achieved by UAE had 92.99% protein purity, higher than those achieved in the control (64.12%) and enzyme groups (77.47%) [32]. No significant effect was appreciated in the current study between the HPP treatment duration (4 and 8 min) and the recovery and purity of the protein extracted from defatted hempseed cake. To our knowledge, there are no similar studies analysing the influence of these HPP parameters on protein extraction; however, similar results were also found in previous studies exploring the extraction of polyphenols from green tea leaves using HPP [33]. Xi et al. [33] explored HPP at multiple pressures (100, 200, 300, 400, 500, and 600 MPa) and times (1, 4, 7, and 10 min) among other extraction conditions and determined that the maximum yield of polyphenols was achieved when using HPP (500 MPa, 1 min), with no further benefits in the yields of polyphenols by increasing the time of the HPP treatments [33]. Casquete et al. [34] suggested that higher phenolic contents and related antioxidant activities were achieved from citrus peels when the samples were HPP pre-treated (300  MPa, 10 min; 500  MPa, 3 min) as compared to the control samples. However, both parameters seemed to decrease when the lemon and orange peels were pre-treated for longer periods of time (500  MPa, 10 min) [34]. Previous research also emphasised that increased pressure in HPP treatments, when used for the purposes of extraction, can modify the properties of the solvent (i.e., increasing strength, solubility, and density of polar compounds) whilst reducing the dielectric constant of water, contributing to decreasing the polarity of the solvent and, thus, favouring the extraction of compounds with lower water solubility, such as flavonols and anthocyanins [35].

### 3.3. Amino Acid Composition of Hempseed Protein Isolates 

UAE had a significant effect (*p* < 0.05) on the levels of EAA, CEAA, NEAA, TAA, and the ratios of EAA/TAA and Arg/Lys in the protein isolates. There was also a significant interaction of UAE*HPP pressure (*p* < 0.05) for all these amino acids, except in the case of EAA, and the interaction of HPP pressure*HPP time (*p* < 0.05) was significant only in the case of the ratio of Arg/Lys. The remaining factors analysed in this study and their interactions were not statistically significant. As seen in the amino acid profile of the protein isolates generated using different technological processing conditions (Table S1), overall, the samples generated using UAE had higher levels of amino acids compared to those extracted using conventional extraction methods, independently of the use or not of HPP pre-treatments. When analysing the ratios of EAA/TAA, these differences were only appreciated between UAE and conventional extraction methods when the duration of the HPP treatment was 8 min and at a pressure of 400 and 600 MPa. In the case of the Arg/Lys ratio, the UAE extraction seems to favour the extraction of Lys over that of Arg, decreasing the Arg/Lys ratios compared to conventional extraction treatments for all the HPP pre-treatments and control (non-pre-treated samples). The slight differences appreciated in the Arg/Lys ratio could also be attributed to the release of free amino acids during UAE extraction [24,36] that are unlikely to be recovered by the isoelectric precipitation process used for the generation of the isolates of this study [37]. However, overall, the results of the current study indicate that UAE had a favourable effect in improving the efficiency of extraction of all amino acids compared to conventional extraction, whether the samples were pre-treated with HPP or not. Further studies are needed in order to estimate the possible loss of free amino acids during isoelectric precipitation that could be further recovered, improving the efficiency of the extraction processes proposed. 

There are few reports analysing the amino acid composition of protein isolates from hemp and the effect of this or any other innovative processing technologies on the final composition of the protein ingredients. Malomo and Aluko [38] determined that the use of 10 kDa ultrafiltration in the preparation of protein ingredients from hempseed meal resulted in higher levels of Val and Leu and lower levels of Arg compared to protein isolates achieved by alkaline extraction–isoelectric precipitation. Moreover, Cabral et al. [17] also appreciated changes in the profile of essential and total amino acids in protein isolates extracted using several solvents, NaOH, KOH, NaHCO_3_, and NaCl. On the other side, Hadnađev et al. [39] determined that the amino acid profile of the protein isolates generated by alkaline extraction followed by isoelectric precipitation and salt extraction followed by membrane filtration had a similar amino acid composition. Overall, the amino acid composition of hempseed proteins has been described as nutritionally valuable as it contains all essential amino acids, although variations in the levels of individual amino acids may occur based on several factors affecting the development of the biomass, including the genotype of hemp and the agronomic conditions [39,40]. The main amino acids of the hempseed protein isolates of this study were Arg, Asp, and Glu, while the first limiting amino acid was Trp, followed by Met and Lys, similar to previous studies [26,40,41].

The amino acid composition data of soya protein isolates and casein, as described by C.-H. Tang et al. [20], that are currently considered nutritional “gold standards” or main proteins representative of “high-quality proteins” from a nutritional perspective is summarised in Table 2. Overall, the levels of all amino acids of hempseed protein isolates in this study were comparable to or a bit lower than those described in soya protein isolates and casein, except in the case of Arg. These results are different from those previously described by C.-H. Tang et al. [20], that generated protein ingredients from defatted hempseed with higher levels of all amino acids, except for tyrosine, valine, leucine, methionine, and lysine, compared to those of casein. These different results could be explained by the inherent biological differences that could be present in the original biomass, as well as by the different methodologies used for the generation of protein isolates that may favour the extraction of different types of proteins in the isolates and, thus, the concentration of different amino acids [38,40]. 

When analysing the ratios of EAA relative to the content of TAA of the isolates (EAA/TAA (%)), the values of the different hempseed protein isolates generated in this study ranged between 30.45 and 32.86%, similar to those described in soya protein isolates, suggesting that these protein isolates are nutritionally beneficial. Similar results comparing the EAA/TAA between hempseed and soya protein isolates were also reported in previous studies emphasizing the nutritional benefits and possible applications of hempseed proteins [40,42]. Furthermore, the ratio of Arg/Lys of the hempseed protein isolates of this study ranged between 3.78 and 5.34, higher than those described in casein and soya and similar to previous reports of hemp protein ingredients [40]. High Arg/Lys ratios have been linked to cardioprotective effects due to their anti-hypercholesterolemic and anti-atherogenic effects [43,44]. This suggests that the hempseed protein isolates generated in this study may be used as a valuable ingredient promoting cardiovascular health deserving of further exploration, especially the role of HPP and UAE in increasing the extraction yields of Lys.

**Figure 3:**
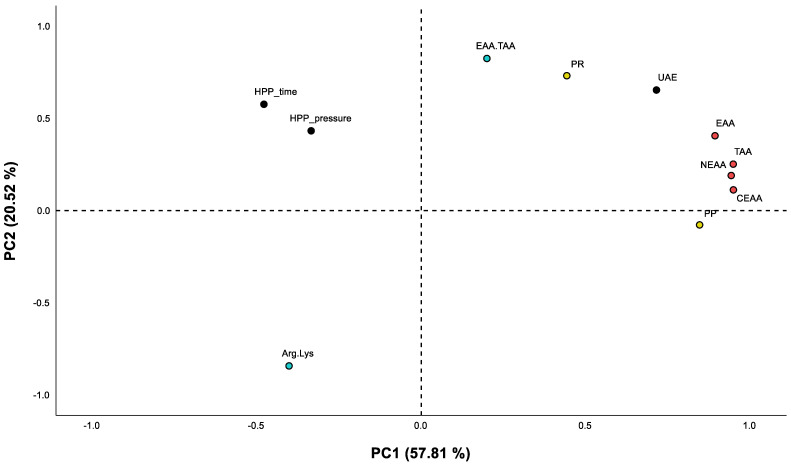
Principal component analysis (PCA) scatter plot representing the scores for the protein extraction (PR—protein recovery and PP—protein purity) and amino acid composition in hempseed protein isolates. Abbreviations in the figure are as follows: EAA (essential amino acids), CEAA (conditionally essential amino acids), NEAA (non-essential amino acids), TAA (total amino acids), EAA.TAA (percentage of essential amino acids from all total amino acids), Arg.Lys (Arg/Lys ratio).

PCA was performed to analyse further the similarities and differences in the protein recovery and purity as well as the amino acid composition and ratios in relation to the main HPP pre-treatment and extraction factors considered in this study (Figure 3). Principal component 1 (PC1) explained 57.81% of the variation of the data set, and PC2 explained 20.52%. PC1 seems to cluster together the application of UAE during the process of extraction with the protein recovery, protein purity, and the EAA, CEAA, NEAA, TAA, and EAA/TAA composition of the protein isolates. PC2 further separates the variation of the data set by separating the Arg/Lys ratio of the protein isolates that seem to be affected differently compared to the other parameters analysed. To our knowledge, there is no literature specific to the effect of HPP combined with UAE on the extraction of these two amino acids, and thus, this effect deserves further investigation if the extracts are intended to be marketed for their cardiovascular benefits based on high Arg/Lys ratios.

### 3.4. SDS-PAGE

**Figure 4:**
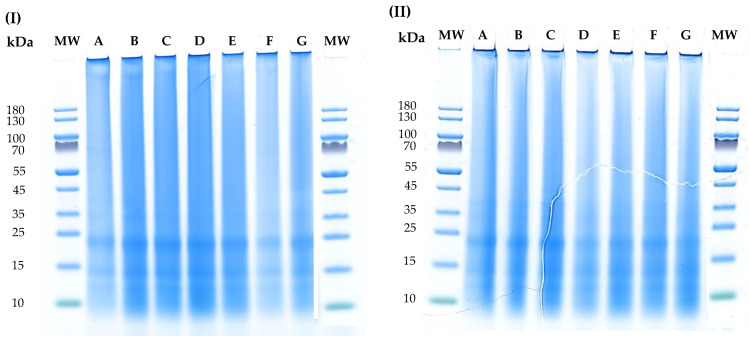
SDS-PAGE gel of hemp meal protein isolates obtained using (**I**) HPP pre-treatments and extracted with conventional method, or (**II**) HPP pre-treatments and extracted with UAE. The lanes: MW—corresponds to the reference protein standard PageRuler pre-stained protein ladder with molecular weight range of 10–180 kDa; lanes A—HPP pre-treatment (600 MPa, 8 min); lanes B—no HPP pre-treatment; lanes C—HPP pre-treatment (200 MPa, 4 min); lanes D—HPP pre-treatment (400 MPa, 8 min); lanes E—HPP pre-treatment (400 MPa, 4 min); lanes F—HPP pre-treatment (600 MPa, 4 min); lanes G—HPP pre-treatment (200 MPa, 8 min).

Previous research characterised the protein from hempseed and identified three main fractions. Edestin is the major protein described in hempseed and can represent 60 to 80% of the total proteins of this biomass. Edestin is composed of an acidic and a basic subunit linked by one disulfide bond with an estimated molecular weight of approximately 300 kDa [23]. The presence of 20 and 33 kDa protein bands corresponding to edestin have been previously described in studies characterising hempseed proteins using SDS-PAGE [20,41,42]. The other two main proteins of hempseed are albumin (up to 20% of the total proteins of hempseed) and other polypeptides [22] that are normally described in SDS-PAGE by molecular weight bands at 18 kDa and 48 kDa, respectively [20,41]. To confirm the molecular weight of the proteins in the hemp protein isolates and to determine whether or not the profiles of the protein isolates were impacted by the various technological processing combinations used, SDS-PAGE studies were carried out. The results of SDS-PAGE can be found in Figure 4. All hemp isolates exhibited a similar protein pattern, where the most distinctive bands were observed at 15 kDa (corresponding to albumin) and around 20 kDa, described as the basic subunit of edestin [41,45]. The absence of bands around 48 kDa and 33 kDa could be explained by the low solubility of those proteins in the isolates. Previous research remarked on the low solubility of hemp meal protein isolates in the electrophoresis buffer, although the pH of the buffer solution is around 8, which is reflected in highly stained although diffused bands in an SDS gel [41]. This can explain the band that was found at the top of the wells, as the insoluble proteins were not able to migrate through the gel. Overall, the proteins of the isolates generated in this study were similar to those previously reported from other protein isolates from hempseed [20]. Moreover, despite the differences in protein extraction (recovery and purity), as well as in the profile of certain amino acids in the isolates, the SDS-PAGE of this study did not allow us to perceive these differences in protein structure. The aforementioned effects of HPP and UAE on the amino acid composition of the protein isolates seem to indicate the need to perform further structural analyses on these isolates to understand the effect of these extraction forces at a molecular level.

## 4. Conclusions

Defatted hempseed meal used in this study had high levels of protein and, thus, the potential to be re-valorised by technological treatments for the generation of protein-rich ingredients (concentrates and isolates). With respect to the evaluation of innovative technologies for the recovery of protein from biomass, overall, HPP followed by UAE achieved the highest protein recovery (≈62%) and purity (≈76%). The amino acids described at high concentrations in the hempseed protein isolates of this study were Arg, Asp, and Glu, while the first limiting amino acid was Trp, followed by Met and Lys, showing the nutritional potential of these isolates and their potential to be included when designing novel food formulations. The extraction methods used had an effect on the composition of these protein isolates, with lower Arg/Lys ratios in UAE processed isolates, while the relative contents of EAA with respect to TAA of the isolates were fairly constant between treatments. All these factors need to be considered further to design nutritionally balanced food products, as food producers will need to know the nutritional attributes of hemp protein isolates to use them to complement and balance the amino acid profile desired in the final formulated food products. However, despite these differences in protein composition, these structural differences were not perceived by SDS-PAGE. Further studies are required to investigate the effects of HPP and UAE on the amino acid composition, specifically the Arg/Lys ratios, the loss of free amino acids during the processes of protein isolation, and the protein structure of these isolates. Additionally, the optimisation of the processing conditions when using these technologies at an industrial scale is necessary to provide a cost estimation of their use and to facilitate the production of food ingredients for their final use as functional foods and nutraceuticals. Elucidating the impact of HPP and UAE on the quality and functionality of protein isolates will enhance our understanding of the potential applications of these technologies in the food industry and their integration into large-scale production processes.

## Figures and Tables

**Table 1:** Proximate composition (moisture, ash, protein, fibre, and crude lipid contents) of the defatted and ground hempseed cake. Results are expressed as average ± standard error of the mean (SEM) (n = 3).

Analytes	Concentration (%) *
Moisture	9.46 ± 0.16
Ash	7.61 ± 0.04
Protein	30.55 ± 0.0
Fibre	34.01 ± 0.64
Crude lipids	6.13 ± 0.77

* Moisture and ash units are % of the analyte in the original biomass; protein, fibre, and crude lipids are expressed as % on a dry weight (DW) basis.

**Table 2:** Amino acid composition (mg/g of protein) of soy protein isolate and casein. Data from this table were reported by C.-H. Tang et al. [20].

AA	Soy PI	Casein
Ala	38.3	27
Arg	75.7	33
Asp	118.1	63
Glu	212.9	190
Gly	38.6	16
His	29	27
Ile	44.8	49
Leu	70	84
Lys	53.9	71
Met	9.3	26
Phe	53	45
Pro	52.9	
Ser	54.8	46
Thr	41	37
Tyr	37.1	55
Val	44.1	60
**EAA**	**345.10**	**399.00**
**CEAA**	**204.30**	**104.00**
**NEAA**	**424.10**	**326.00**
**TAA**	**973.50**	**829.00**
**EAA/TAA (%)**	**35.45**	**48.13**
**Arg/Lys**	**1.40**	**0.46**

Abbreviations in the table are as follows: AA (amino acid), EAA (essential amino acids), CEAA (conditionally essential amino acids), NEAA (non-essential amino acids), TAA (total amino acids), and PI (protein isolate).

## Data Availability

Experimental data pertinent to this study can be available from the corresponding author upon reasonable request.

## Supplementary Materials

The following supporting information can be downloaded at: https://www.mdpi.com/article/10.3390/foods12152883/s1, Table S1: Amino acid composition of protein extracts generated from hempseed cake using different combinations of high-pressure processing (HPP) pre-treatments (time and pressure) and extraction methods (conventional extraction (CE), and ultrasound-assisted extraction (UAE)). Results are expressed as average ± standard error of the mean (SEM) (n = 4).
