# Supplementary material for: Recovery of Protein from Industrial Hemp Waste (Cannabis sativa, L.) Using High-Pressure Processing and Ultrasound Technologies
Source: Foods. 2023 Jul 29;12(15):2883. doi: 10.3390/foods12152883 (PMC10417828; doi:10.3390/foods12152883)
Supplement: Supplementary file 1 [file foods-12-02883-s001.zip › foods-2492596-supplementary.pdf]

Table S1. Amino acid composition of protein extracts generated from hempseed cake using different combinations of high-pressure processing (HPP) pre-treatments (time and pressure) and extraction methods (conventional extraction (CE), and ultrasound-assisted extraction (UAE)). Results are expressed as average ± standard error of the mean (SEM) (n=4).

**HPP pre-treatments**														
**Control (no HPP pre-treatment)**			**200 MPa**				**400 MPa**				**600 MPa**			
**AA**	**Extraction**		**HPP (min)**	**AA**	**Extraction**		**HPP (min)**	**AA**	**Extraction**		**HPP (min)**	**AA**	**Extraction**	
	**CE**	**UAE**			**CE**	**UAE**			**CE**	**UAE**			**CE**	**UAE**
Ala	29.63±0.35	37.99±0.41		Ala	29.02±0.30	35.82±0.23		Ala	29.87±0.40	34.10±0.61		Ala	28.62±1.07	37.53±1.47
Arg	73.42±2.08	85.42±1.15		Arg	71.23±2.27	80.75±0.29		Arg	68.31±3.19	68.68±2.04		Arg	68.56±3.75	81.43±3.02
Asx	77.63±2.67	96.46±0.63		Asx	76.96±1.63	86.19±0.50		Asx	72.66±0.98	82.09±4.64		Asx	70.25±3.74	92.72±3.37
Glx	129.40±3.50	154.16±1.02		Glx	124.44±2.67	146.26±0.57		Glx	122.32±5.98	132.46±4.64		Glx	119.07±7.23	154.03±10.22
Gly	29.04±0.47	36.53±0.43		Gly	28.27±0.50	35.03±0.28		Gly	29.21±0.60	33.07±0.61		Gly	28.44±1.15	35.43±1.26
His	16.08±0.59	20.73±0.46		His	15.97±0.27	18.82±0.06		His	15.22±0.22	17.67±0.69		His	14.60±0.76	19.69±0.84
Ile	22.71±0.62	28.68±0.82		Ile	23.06±0.42	26.35±0.38		Ile	22.07±0.40	26.03±0.75		Ile	21.35±1.07	28.99±1.41
Leu	45.72±0.61	57.16±0.56		Leu	44.62±0.32	54.98±0.26		Leu	45.74±0.53	52.33±1.04		Leu	43.86±1.78	56.61±2.42
Lys	13.84±0.19	19.59±0.29		Lys	13.34±0.14	19.85±0.19		Lys	14.11±0.30	18.15±0.05		Lys	13.56±0.57	19.56±0.60
Met	2.84±0.06	5±1.19		Met	3.25±0.25	6.87±0.29		Met	3.63±0.15	6.08±0.98		Met	2.62±0.12	6.07±0.32
Phe	31.37±0.69	38.85±0.41		Phe	31.01±0.38	36.15±0.10		Phe	30.11±0.54	34.47±1.06		Phe	28.75±1.60	38.51±1.66
Pro	22.16±3.65	32.36±2.11		Pro	21.44±3.92	31.01±1.64		Pro	26.15±0.85	30.66±1.30		Pro	22.84±5.10	28.68±3.63
Ser	33.93±0.68	42.11±0.39		Ser	32.92±0.68	40.02±0.27		Ser	32.68±1.09	35.16±0.90		Ser	32.03±1.42	40.35±1.39
Thr	19.94±0.64	24.72±0.52	4	Thr	19.66±0.40	22.90±0.17	4	Thr	19.07±0.41	19.84±0.56	4	Thr	18.40±0.91	23.69±0.85
Trp	1.39±0.08	2.44±0.19		Trp	1.15±0.26	2.13±0.06		Trp	1.41±0.20	1.75±0.15		Trp	0.96±0.14	1.79±0.42
Tyr	25.41±0.56	31.80±0.31		Tyr	24.69±0.20	29.63±0.05		Tyr	24.49±0.51	28.18±0.74		Tyr	23.43±1.23	31.29±1.42
Val	30.71±0.84	38.52±0.80		Val	30.62±0.30	35.00±0.34		Val	29.78±0.58	35.24±1.24		Val	28.57±1.46	39.09±1.82
**EAA**	**184.61±4.02 b**	**235.70±2.11 a**		**EAA**	**182.68±2.41 b**	**223.03±0.30 a**		**EAA**	**181.14±2.42 b**	**211.56±6.44 a**		**EAA**	**172.68±7.87 b**	**234.00±10.23 a**
**CEAA**	**150.03±4.22 b**	**186.11±2.39 a**		**CEAA**	**145.62±6.17 b**	**176.43±1.96 a**		**CEAA**	**148.15±3.69 b**	**160.59±3.36 a**		**CEAA**	**143.28±9.56 b**	**176.82±8.72 a**
**NEAA**	**270.58±6.52 b**	**330.71±1.86 a**		**NEAA**	**263.35±3.90 b**	**308.28±1.43 a**		**NEAA**	**257.53±8.19 a**	**283.80±9.44 a**		**NEAA**	**249.97±13.11 b**	**324.63±16.43 a**
**TAA**	**605.22±11.34 b**	**752.52±5.38 a**		**TAA**	**591.65±9.61 b**	**707.74±2.78 a**		**TAA**	**586.82±13.20 b**	**655.95±19.04 a**		**TAA**	**565.93±29.11 b**	**735.46±35.09 a**
**EAA/TAA (%)**	**30.50±0.29 a**	**31.32±0.20 a**		**EAA/TAA (%)**	**30.89 ± 0.53 a**	**31.51±0.14 a**		**EAA/TAA (%)**	**30.90±0.53 a**	**32.25±0.12 a**		**EAA/TAA (%)**	**30.55±0.53 a**	**31.84±0.21 a**
**Arg/Lys**	**5.30±0.10 a**	**4.36±0.08 b**		**Arg/Lys**	**5.34±0.21 a**	**4.07±0.03 b**		**Arg/Lys**	**4.84±0.19 a**	**3.78±0.11 b**		**Arg/Lys**	**5.06±0.17 a**	**4.16±0.09 b**

8	Ala	28.55±0.24	34.58±0.19	8	Ala	28.30±0.70	34.85±0.64	8	Ala	27.97±0.62	35.34±1.36
	Arg	68.31±1.53	71.03±1.49		Arg	68.20±3.22	71.64±1.59		Arg	67.73±2.25	74.88±3.86
	Asx	71.08±0.96	81.74±1.03		Asx	70.61±2.25	83.10±3.22		Asx	70.36±2.55	88.02±4.54
	Glx	119.11±2.15	134.54±0.62		Glx	119.55±6.83	136.50±3.27		Glx	117.63±2.98	143.92±8.55
	Gly	27.77±0.25	33.84±0.25		Gly	28.29±0.61	33.23±0.36		Gly	27.24±0.66	34.41±1.04
	His	14.97±0.13	17.87±0.15		His	14.92±0.62	18.02±0.33		His	14.68±0.39	18.58±0.79
	Ile	21.98±0.23	25.65±0.13		Ile	21.34±0.58	26.44±0.49		Ile	21.55±0.52	26.58±1.52
	Leu	44.14±0.30	53.11±0.22		Leu	43.48±1.30	53.48±0.72		Leu	43.13±0.94	54.04±2.29
	Lys	13.68±0.14	18.78±0.43		Lys	13.55±0.30	18.41±0.44		Lys	13.58±0.20	19.26±0.83
	Met	2.81±0.12	4.95±0.14		Met	2.46±0.29	8.69±2.15		Met	2.72±0.14	6.98±1.85
	Phe	29.78±0.23	34.63±0.33		Phe	28.96±1.17	35.66±0.77		Phe	29.25±0.61	35.81±1.91
	Pro	21.33±3.50	30.64±1.90		Pro	25.31±1.35	24.02±3.70		Pro	22.94±0.67	31.80±1.83
	Ser	31.57±0.48	36.18±0.83		Ser	32.23±1.18	36.40±0.48		Ser	31.46±0.77	38.71±1.65
	Thr	18.48±0.25	20.02±0.72		Thr	18.75±0.64	20.71±0.40		Thr	18.36±0.54	22.29±1.29
	Trp	1.02±0.15	2.01±0.02		Trp	1.04±0.21	2.69±0.26		Trp	1.11±0.10	1.98±0.28
	Tyr	23.98±0.23	28.36±0.26		Tyr	23.33±0.95	28.80±0.67		Tyr	23.47±0.44	29.39±1.43
	Val	29.37±0.28	34.01±0.30		Val	28.63±0.71	35.45±0.61		Val	28.70±0.55	35.72±2.10
	**EAA**	**176.23±1.24 b**	**211.03±1.97 a**		**EAA**	**173.14±5.41 b**	**219.56±3.66 a**		**EAA**	**173.08±3.65 b**	**221.24±12.76 a**
	**CEAA**	**141.39±4.44 b**	**163.87±2.81 a**		**CEAA**	**145.12±3.64 b**	**157.69±3.98 a**		**CEAA**	**141.38±4.00 b**	**170.47±7.08 a**
	**NEAA**	**250.31±3.67 b**	**287.04±2.12 a**		**NEAA**	**250.69±10.94 b**	**290.88±7.47 a**		**NEAA**	**247.42±6.50 b**	**305.99±16.06 a**
	**TAA**	**567.94±7.25 b**	**661.94±6.48 a**		**TAA**	**568.96±19.64 b**	**668.13±9.60 a**		**TAA**	**561.88±14.07 b**	**697.70±35.80 a**
	**EAA/TAA (%)**	**31.04±0.32 a**	**31.88±0.13 a**		**EAA/TAA (%)**	**30.45±0.30 b**	**32.86±0.23 a**		**EAA/TAA (%)**	**30.81±0.14 b**	**31.68±0.21 a**
	**Arg/Lys**	**4.99±0.07 a**	**3.78±0.01 b**		**Arg/Lys**	**5.03±0.13 a**	**3.89±0.05 b**		**Arg/Lys**	**4.99±0.12 a**	**3.88±0.06 b**

Different lower-case letters indicate statistical differences ($P<0.05$) amongst samples extracted following CE and UAE conditions within each pre-treatment condition used. Abberviations in the table are as follows: EAA (essential amino acids), CEAA (conditionally essential amino acids), NEAA (non-essential amino acids) and TAA (total amino acids).
